# Math Anxiety Mediates the Link Between Number Sense and Math Achievements in High Math Anxiety Young Adults

**DOI:** 10.3389/fpsyg.2020.01095

**Published:** 2020-05-26

**Authors:** Paula Andrea Maldonado Moscoso, Giovanni Anobile, Caterina Primi, Roberto Arrighi

**Affiliations:** Department of Neuroscience, Psychology, Pharmacology and Child Health, University of Florence, Florence, Italy

**Keywords:** approximate number sense (ANS), numerical cognition, math anxiety, math abilities, Weber fraction

## Abstract

In the past few years, many studies have suggested that subjects with high sensory precision in the processing of non-symbolic numerical quantities (approximate number system; ANS) also have higher math abilities. At the same time, there has been interest in another non-cognitive factor affecting mathematical learning: mathematical anxiety (MA). MA is defined as a debilitating emotional reaction to mathematics that interferes with the manipulation of numbers and the solving of mathematical problems. Few studies have been dedicated to uncovering the interplay between ANS and MA and those have provided conflicting evidence. Here we measured ANS precision (numerosity discrimination thresholds) in a cohort of university students with either a high (>75th percentile; *n* = 49) or low (<25th percentile; *n* = 39) score on the Abbreviate Math Anxiety Scale (AMAS). We also assessed math proficiency using a standardized test (MPP: Mathematics Prerequisites for Psychometrics), visuo-spatial attention capacity by means of a Multiple Objects Tracking task (MOT) and sensory precision for non-numerical quantities (disk size). Our results confirmed previous studies showing that math abilities and ANS precision correlate in subjects with high math anxiety. Neither precision in size-discrimination nor visuo-spatial attentional capacity were found to correlate with math capacities. Interestingly, within the group with high MA, our data also revealed a relationship between ANS precision and MA, with MA playing a key role in mediating the correlation between ANS and math achievement. Taken together, our results suggest an interplay between extreme levels of MA and the sensory precision in the processing of non-symbolic numerosity.

## Introduction

Numerical and mathematical competencies are central predictors of an individual’s success in life. Developing adequate numerical and mathematical skills is a prerequisite to accomplishing numerous tasks in daily life, such as setting and keeping to a budget (Parsons and Bynner, 2005), as well as pursuing careers in the STEM fields: science, technology, engineering, and mathematics (STEM; Beilock and Maloney, 2015; Ferguson et al., 2015). Impairments in mathematical skills might be triggered by several factors and, amongst these, mathematical anxiety (MA) has been suggested to play a key role. MA has been defined as feelings of apprehension and increased physiological reactivity when individuals have to manipulate numbers, solve mathematical problems, or when they are exposed to an evaluative situation connected to math (Hembree, 1990; Ashcraft, 2002). Similar to other performance-based anxieties, MA involves psychological arousal, negative cognitions, escape and/or avoidance behaviors and, when the individual cannot avoid the situation, performance deficits. MA is also related to reduced cognitive reflection (Morsanyi et al., 2014; Primi et al., 2018), and poorer decision making performance (e.g., Rolison et al., 2016). In other words, MA is described as a multidimensional construct that is related to, but distinct from, other forms of anxiety, such as trait, social, or test anxiety (Ashcraft and Moore, 2009; Vukovic et al., 2013). MA has been shown to hinder math performance. It has been reported that individuals with higher levels of MA obtain lower scores in math achievement tests, take fewer math courses, and tend to avoid career paths involving mathematics (Ma, 1999; Ashcraft and Krause, 2007; Ashcraft and Moore, 2009).

Two theoretical frameworks have traditionally been proposed to account for the link between MA and math achievements (Carey et al., 2016). The deficit theory posits that poor mathematical performance leads to future high levels of MA. In line with that, it has been suggested that MA could result from low numerical (and/or spatial) skills which compromise the development of high proficiency in mathematical problem solving (Maloney et al., 2011; Maloney, 2016). On the other hand, the cognitive interference theory posits that it is MA that affects subsequent mathematical performance. During the phases of information processing and recall, MA would create cognitive interference which affects mathematical performance. According to this theory, anxiety would generate intrusive thoughts to reduce working memory (WM) capacity, with these thoughts acting as a secondary task draining resources that, otherwise, would have been allocated to solving the mathematical task (Ashcraft and Kirk, 2001). An alternative theory posits that MA and mathematical performance show a bidirectional relationship (Ashcraft and Krause, 2007); past failures and negative experiences in mathematical performance would lead to MA which, subsequently, would lead to poorer mathematical performance and vice versa (Ma and Xu, 2004).

Whatever the nature of the link between MA and low achievement in math learning, several studies have highlighted various factors that might account for the negative relationship between these factors. A possible explanation of the gap in math performance between students with high and low levels of MA derives from behavioral and psychophysiological studies, which provide converging evidence for individual (cognitive, affective/physiological, motivational) and environmental (social/contextual) factors (Chang and Beilock, 2016). Recent reports, focused on genetic and neurophysiological factors, suggested that MA arises from a basic level deficiency in symbolic numerical processing. In particular, genetic studies of MA in twins evidenced that genetic factors accounted for about 40% of the variation in MA, and that 12% of the total variance in MA was associated with genetic influences related to math problem-solving (Wang et al., 2014; Malanchini et al., 2017). Finally, children with high mathematical anxiety (HMA), compared with low mathematical anxiety (LMA) peers, show reduced responses in posterior parietal cortex, including the intraparietal sulcus (IPS) and dorsolateral prefrontal cortex regions, known to play a critical role not only in numerical and mathematical cognition, but also in non-symbolic number evaluation (Dehaene et al., 1999; Eger et al., 2003; Piazza et al., 2004; Young et al., 2012; Castaldi et al., 2016).

Whilst symbolic numerical representation and arithmetic are recent cultural inventions specifically adopted by humans, humans share with many non-human animal species an intuitive “approximate number system” (ANS), which is the core ability to automatically and efficiently process numerical magnitude information (Dehaene, 2011). The sensory precision of this system is refined during development and varies considerably between individuals (Halberda et al., 2008; Halberda et al., 2012; Odic et al., 2013). It is suggested that numerosity represents a primary visual attribute (Anobile et al., 2016b) and, in line with this idea, recent studies showed that numerosity is spontaneously perceived, even by 5-year old children (Cicchini et al., 2016). Interestingly, several studies reported strong correlations between the precision in numerosity judgments and current, future or past formal mathematical skills in children (Halberda et al., 2008; De Smedt et al., 2009; Anobile et al., 2013, 2018a; Feigenson et al., 2013; Starr et al., 2013). Complementary studies carried out on subjects with mathematical disabilities (developmental dyscalculia) show that a deficit in mathematical processing generalizes to yield severe difficulties in estimating and comparing numerosity (Landerl et al., 2004; Piazza et al., 2010; Mazzocco et al., 2011; Pinheiro-Chagas et al., 2014; Anobile et al., 2019b). In light of all these results, some authors suggested that an intact number sense might be a base prerequisite for the later mathematical acquisition or, in other words, that the number sense acted as an early non-symbolic start-up tool for the later development of language-based formal mathematical skills (Butterworth, 1999; Piazza, 2010; Butterworth et al., 2011; Dehaene, 2011).

Given the intimate relationship between MA and mathematical achievements, and the complementary link between these and the ANS, it has also been suggested that there is a possible interplay between ANS and MA. However, evidence collected so far is controversial. In particular, two studies have found that individuals with HMA represent numerical magnitude less precisely than their LMA peers (Maloney et al., 2011; Núñez-Peña and Suárez-Pellicioni, 2014). However, as both studies tested with Arabic digits, they only supported a link between MA and symbolic representation of quantity, not numerosity. Recently Braham and Libertus (2018) showed that the association between precision in perceived numerosity (ANS acuity) and subjects’ performance in applied problem solving was present only in subjects with HMA levels, suggesting that an efficient ANS system might act as a potential protective factor for highly math anxious students. Another study reported a link between non-symbolic numerical processing and MA (Lindskog et al., 2017); these authors found that people with high levels of math anxiety show poorer precision in a non-symbolic numerical comparisons task, compared to those with low levels of math anxiety. They also showed that the correlation between math skills and numerosity precision was fully mediated by participants’ level of MA. However, several studies measuring ANS acuity by means of non-symbolic tasks failed to find a significant correlation between ANS and MA in both adults (Dietrich et al., 2015; Colomé, 2019) as well as children (Gómez-Velázquez et al., 2015; Wang et al., 2015; Hart et al., 2016), leaving open the question of whether this interplay occurs.

The current study aims to assess the role of MA in math skills and numerosity perception. We devised two groups with extremely low or high levels of mathematical anxiety (drawn from a large sample of university students) and measured, in both groups, differences in ANS acuity and math abilities as well as correlations between these variables. We first investigated whether the numerosity thresholds were different in subjects with HMA compared to their LMA peers. Then we addressed the question whether any possible numerosity impairments in HMA participants ware selective for numerosity or whether it was related to a more general perceptual weakness in magnitude judgments. This goal was achieved by measuring discrimination thresholds on a non-numerical magnitude task, in which participants were engaged in an object-size discrimination task. The issue of specificity was also tested by measuring a non-magnitude parietal function, as many studies suggested a key role of parietal cortex in both numerosity perception and math processing. To this aim, we decided to administer a Multiple Object Tracking (MOT) task as it was shown to activate the parietal cortex, which has been found to correlate well with both numerosity and math abilities (Corbetta and Shulman, 2002; Ansari et al., 2007; Steele et al., 2012; Anobile et al., 2013). In order to assess the specific role played by MA in mathematical performance, we measured individuals’ anxiety on a more general dimension, such as performance anxiety (Ashcraft and Ridley, 2005; Lindskog et al., 2017). Finally, we tested for the potential mediation role of MA on the link between ANS and math abilities, using a mediation model in which ANS was associated with math achievement through math anxiety. Mediation implies a situation where the effect of the independent variable (*X*) on the dependent variable (*Y*) can be explained using a third mediator variable (*M*) which is caused by the independent variable and is itself a cause for the dependent variable. By modeling an intermediate variable, the overall effect between *X* and *Y* can be decomposed into component parts called the direct effect of X on Y and the indirect effect of X on Y through M (i.e., the mediated effect).

The importance of our study, which took into consideration several possible differences between subjects with high and low math anxiety, relies on the fact that such multidimensional analysis is the most suitable tool to investigate the effect of MA on both low-level quantity processing (ANS) as well as high-level mathematical proficiency. Such an approach is not only likely to allow a full understanding of the interplay between MA, math achievements and ANS, but will also improve understanding of the brain mechanisms underpinning these processes, as well as providing useful information about how to optimize mathematical learning procedures or customized early targeted interventions.

## Materials and Methods

### Participants

Participants were 88 university students attending an introductory statistics course at the School of Psychology of the University of Florence. They were selected from a class of 179 students based on their level of math anxiety. The LMA group comprised 39 participants (69% female; age range 18–22 years, mean = 20.1, *SD* = 0.7) who scored below the 25th percentile (score range 10–19, mean = 16.3, *SD* = 2.6) on the Abbreviated Math Anxiety Scale (AMAS; Hopko et al., 2003). The HMA group comprised 49 participants (82% female; age range 18–37, mean = 20.4, *SD* = 2.9) who scored above the 75th percentile on the AMAS (score range 27–40, mean = 30.1, *SD* = 3.2). All students participated on a voluntary basis. The whole procedure was performed in accordance with the declaration of Helsinki.

### Measures

The *Mathematics Prerequisites for Psychometrics* (MPP; Galli et al., 2011) is a test which was developed to measure the mathematical skills of students enrolled in statistics courses. The scale was developed using item response theory (IRT) because it offers a different value of test precision for each specific level of underlying latent variable being measured, and it does not assume that a single estimate of reliability, and corresponding standard error of measurement, is sufficient to describe precision of measurement over all levels of ability (Embretson and Reise, 2000). The scale consists of 30 problems and has a multiple-choice format (one correct response out of four options). For example, “The value 0.05 is” (i) lower than 0; (ii) between − 1 and 0; (iii) higher than 0.1; and (iv) between 0 and 1, and “Knowing that xy = 3 which of the following is true?” (i) y = 3/x; (ii) y = 3x; (iii) c = 3x; and (iv) xy/3. The sum of correct responses gave us a single composite score for each participant. In the present sample, Cronbach’s α was 0.73 (IC:0.70–0.78). We used this measure as an estimate of the students’ math knowledge (Primi et al., 2014).

The *Abbreviated Math Anxiety Scale* (AMAS; Hopko et al., 2003; Italian version: Primi et al., 2014) measures MA experienced by students in learning and test situations. Participants were required to respond on the basis of how anxious they would feel during given events (for example, “Listening to another student explain a math formula” or “Starting a new chapter in a math book”) by using a 5-point response scale (ranging from strongly agree to strongly disagree). High scores on the scale indicate HMA. A single composite score was obtained, based on participants’ ratings of each statement. In the present sample, Cronbach’s α was 0.84 (IC:0.80–0.87).

The *Test Anxiety Inventory* (TAI; Spielberger et al., 1978) was developed to measure anxiety associated with task-performing situations in high school and college students. The test consists of 20 items, which investigate a range of anxiety symptoms occurring before, during or after exams. Responses are collected using a 4-point Likert scale ranging from 1 (almost never) to 4 (always). The TAI yields a total score calculated as the sum of all 20 items, with higher scores corresponding to high test anxiety. In the present sample, Cronbach’s α was 0.94 (IC:0.93–0.96).

#### Numerosity Discrimination Task

**FIGURE 1:**
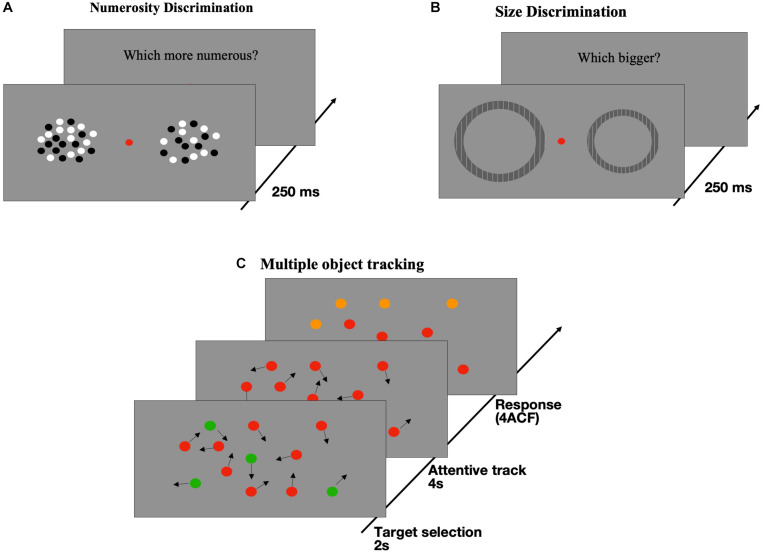
Illustration of tasks and stimuli. **(A)** *Numerosity Discrimination:* two patches of dots were briefly (250 ms) presented to both side of a central fixation point. Subjects were required to select which dots ensemble was more numerous. **(B)** *Size Discrimination:* Participants were asked to indicate which of two briefly (250 ms) presented annuli was perceived as being larger (method adapted from Pooresmaeili et al., 2013). **(C)** *Multiple Object Tracking (MOT):* At the beginning of the session, some disks (2, 3, or 4) out of twelve were colored in green with the remaining being red. All dots moved randomly on the screen (7°/s) for a period of 2 s then the green disks turned red (like the distracters) and subjects had to track them for 4 s. At the end of the tracking period, all dots stopped and 4 of them turned orange with one of the orange dots being green at the beginning. This dot was the target subjects had to indicate in a 4-alternative forced paradigm (4ACF).

Stimuli consisted of two brief (250 ms) patches of dots, presented on either side of a central fixation point (Figure 1A). Dots were 0.25° in diameter, half white and half black (to balance luminance), presented at 80% contrast on a gray background of 40 cd/m^2^. They were constrained to fall within a virtual circle of 10° diameter, centered at 10° eccentricity. Standard numerosity (randomly left or right) was fixed at 24 dots while the probe adaptively changed, according to participant responses, with numerosity defined by an adaptive staircase QUEST algorithm (Watson and Pelli, 1983). All participants performed one session of 80 trials. Participants were asked to indicate the side of the screen with more dots. We plotted the proportion of trials where the standard stimulus appeared more numerous than the probe against the probe numerosity (on log axis) and fitted with cumulative Gaussian error functions. We defined the point of subjective equality (PSE) as the physical numerosity of the probe yielding 50% of probe more numerous responses. Then we defined subjects’ precision as just notable difference (JND), that is the numerosity offset defining the 50–75% range of probe more numerous. Finally, normalizing PSE by JND we obtained a single index Weber Fraction (WF), a typical dimensionless psychophysical index for discrimination thresholds.

#### Size Discrimination Task

Stimuli were gratings sinusoidally modulated in luminance with a spatial frequency of 2 cycles per degree and a Michelson contrast of 90% which were vignetted in an annular contrast window (Figure 1B). In each trial, two annuli were simultaneously presented for 250 ms on the left and the right side of the central fixation point, at an eccentricity of 10°. Subjects were required to indicate which stimulus appeared to be larger. The diameter of the test stimulus (presented randomly on the left or right) was 5° or 8° (40 trials each, randomized trial-by-trial), while the probe varied in diameter by a percentage drawn randomly from a Gaussian distribution centered at 0 with *SD* = 20%. To minimize alternative judging strategies (such as estimating border-to-center of the screen distance), we independently jittered the horizontal eccentricity of the test and the probe between 8.5° and 11.5°, and their distance from the horizontal meridian within ± 3°. After the stimuli presentation, a 100 ms full-screen random noise mask was displayed to cancel out possible afterimages. The proportion of “test largest” trials was plotted against the log-ratio of the test to probe and fitted with cumulative Gaussian error functions. Even for the size discrimination task, the dependent variable which we took into account was Weber Fraction (see above), indicating subjects’ sensory precision in the size discrimination thresholds.

#### Visual Sustained Attention Task

Visual sustained attention (Figure 1C) was measured by a multiple object tracking task (MOT; Pylyshyn and Storm, 1988). At each trial, a total of twelve disks with a diameter of 0.9° moved randomly on the full screen at 7°/s for a period of 2 s. The green targets could be 2, or 3, or 4 (representing the three conditions) and the remaining stimuli (distractors) were red. After the 2 s, the green targets turned red (like the distractors), and continued to move randomly on the full screen for 4 s. The participants were required to continue to track them with their attention. After this period, the disks stop moving, and 4 of them turned orange. Participants had to identify (using the mouse cursor) which one of the four orange items was a green target at the beginning of the trial (4AFC). Each experimental session had 10 trials and participants performed 2 sessions, for a total of 20 trials. No feedback was provided. We measured the performance of the participants as the proportion of correct responses for each condition (Anobile et al., 2013).

### Procedure

Participants were tested individually. Before the testing sessions, students provided informed consent. Math skills (MPP), Math anxiety (AMAS), and Test anxiety (TAI) were all measured before psychophysical experiments. The scales were in a paper-and-pencil format. The psychophysical tasks were then performed in a quiet and dimly illuminated room. Participants sat in front of a BARCO 27” monitor subtending 39° by 29° from the subject’s viewing distance of 57 cm. The monitor resolution was 1024 × 768 and the refresh rate equal to 120 Hz. Stimuli for the psychophysical experiments were all generated and presented with PsychToolbox (Brainard, 1997) routines for MATLAB (ver. 2010a, The Mathworks, Inc.).

### Statistical Analysis

Preliminarily, we tested differences within the group (LMA and HMA) on numerosity and size discrimination tasks as well as sustained attention with a mixed 3 (within factor: tasks) × 2 (between factor: groups) ANOVA. Correlations between variables were tested by Pearson’s r. To further enhance the understanding of the mechanisms underlying the relationships among these variables, a mediation model was tested. Specifically, MA was modeled as the intermediate variable (M) between ANS and math proficiency. This procedure allowed us to conclude whether the independent variable influences the dependent variable directly (path c’ in Figure 5) and/or indirectly (path a or b in Figure 5) through the mediator. Obviously, the direct and indirect effects added to the yield of the total effect (path c in Figure 5) of the independent variable on the dependent variable. The mediation model was estimated to derive from the total, direct, and indirect effects of ANS on math achievement through MA. The indirect effect of ANS on math achievement was quantified as the product of the ordinary least squares (OLS) regression coefficient estimating MA from ANS (i.e., path a in Figure 5) and the OLS regression coefficient estimating math achievement from MA when controlling for ANS (i.e., path b in Figure 5). To test the mediation model, we used the INDIRECT macro for SPSS (Hayes, 2013). The INDIRECT macro tested the hypothesized model using a bootstrapping procedure (with 5000 bootstrap samples) to estimate the 95% confidence interval for the indirect (mediated) effect (for more details, see Preacher and Hayes, 2008). Bootstrapping is a resampling strategy for estimation and hypothesis testing. With the bootstrapping method, the sample is conceptualized as a pseudo-population that represents the broader population from which the sample was derived, and the sampling distribution of any statistic can be generated by calculating the statistic of interest in multiple resamples from the dataset. The bootstrapping procedure has been suggested as representing the most trustworthy test for assessing the effects of mediation models, overcoming issues associated with inaccurate *p*-values which result from violations of parametric assumptions (Hayes and Scharkow, 2013). Indeed, the bootstrapping procedure is advantageous because it does not impose the assumption of normality on the sampling distribution of indirect effects, and it retains high power while maintaining adequate control over Type I error rate (MacKinnon et al., 2002; Mackinnon et al., 2004; Preacher and Hayes, 2008; Hayes, 2009). The bootstrap test is statistically significant (at 0.05) if both confident limits have the same sign (e.g., both positive and both negative). This indicates that zero is not a likely value, and therefore, that the null hypothesis of a null indirect effect has to be rejected.

## Results

### Differences Between Groups

**FIGURE 2:**
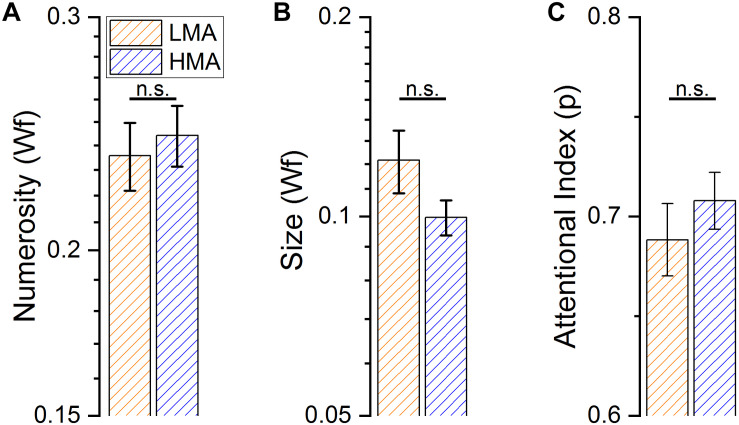
Performance in the three different psychophysical tasks. **(A)** Average numerosity discrimination thresholds (Weber fraction) for subjects with high (HMA) and low (LMA) levels of math anxiety. **(B)** Average object-size discrimination thresholds (Weber fraction) for subjects with high (HMA) and low (LMA) levels of math anxiety. **(C)** Average proportion of correct response in the Multiple Object Tracking task, for subjects with high (HMA) and low (LMA) levels of math anxiety.

At first, we measured the difference in math anxiety between the students in the HMA and LMA group that turned out in being highly statistically significant [*t*(86) = -21.85, *p* < 0.001]. We then measured performance difference between HMA and LMA groups in the psychophysical tasks (see Table 1 for descriptive statistics). Numerosity and size discrimination thresholds (WF) were measured separately for each participant. Attentional performance in the MOT task was computed as a percentage of correct responses separately for the three experimental conditions (tracking of 2, 3 or 4 dots) however, given all these conditions turned out to be highly correlated to each other (Mot 2 and Mot 3 *r* = 0.351, *p* < 0.001; Mot 2 and Mot 4 *r* = 0.305, *p* = 0.004; Mot 3 and Mot 4 *r* = 0.61, *p* < 0.0001), we computed a single index to estimate the performance in the attentional task by averaging the scores across conditions. Individuals in the low and high math-anxiety groups, showed similar performance across all tasks [*F*(1, 86) = 0.036, *p* = 0.85]; the interaction was also not significant [*F*(2, 172) = 1.539, *p* = 0.218]. *Post hoc t*-test confirmed the differences between groups were not significant in both, numerosity and size discrimination tasks [Numerosity Wf: *t*(86) = −0.444, *p* = 0.658; Size Wf: *t*(86) = 1.607, *p* = 0.112, Figures 2A,B]. Similarly, performance in the attentional task did not turn out to be statistically significant between the two groups considering neither the aggregate index (Figure 2C), nor each experimental condition (defined by the number of objects to track) independently [Mot 2: *t*(86) = -0.24, *p* = 0.8; Mot 3: *t*(86) = -1.95, *p* = 0.05; Mot 4: *t*(86) = 0.28, *p* = 0.78]. Finally, not only the LMA group had statistically higher math proficiency but also lower test anxiety scores compared to the HMA group [*t*(85) = 2.923, *p* = 0.004; *t*(85) = -8.75, *p* < 0.001 for math performance and test anxiety score respectively].

**TABLE 1:** Descriptive statistics for LMA and HMA groups.

	LMA			HMA		
	*M*	*SD*	*N*	*M*	*SD*	*N*
ANS Wf (%)	23.57	8.69	39	24.41	9.02	49
Size Wf (%)	12.15	8.26	39	9.96	4.27	49
Attentional Index	0.69	0.11	39	0.71	0.1	49
Math performance	23.63	3.51	38	21.33	3.75	49
Math anxiety	16.36	2.57	39	30.08	3.18	49
Test anxiety	34.34	9.42	38	55.20	12.12	49

### Correlations Between Variables

After showing that the two math-anxiety groups did not differ in their precision to discriminate stimuli numerosity or size and were also comparable in terms of attentional performance, we investigated the relationships between perceptual and non-perceptual measures within the two groups (see Table 2 for full correlation values).

**TABLE 2:** Pearson correlations between all measured variables in the HMA sub-group (above diagonal) and LMA sub-group (below diagonal).

**Measure**	**1**	**2**	**3**	**4**	**5**	**6**
1. Math performance	–	−0.290*	–0.186	–0.014	−0.479***	–0.009
2. ANS acuity	–0.205	–	–0.062	–0.082	0.481***	0.073
3. Size acuity	–0.139	–0.023	–	0.128	–0.065	–0.156
4. Attentional index	0.242	−0.330*	−0.297*	–	−0.255*	–0.212
5. Math anxiety	–0.261	0.073	0.140	–0.256	–	0.104
6. Test anxiety	0.087	–0.008	–0.108	0.047	0.072	–

* *p* < 0.05, *** *p* < 0.001. Bold numbers indicate significant correlations.

For clarity, we will describe the data separately for the two math-anxiety groups.

**FIGURE 3:**
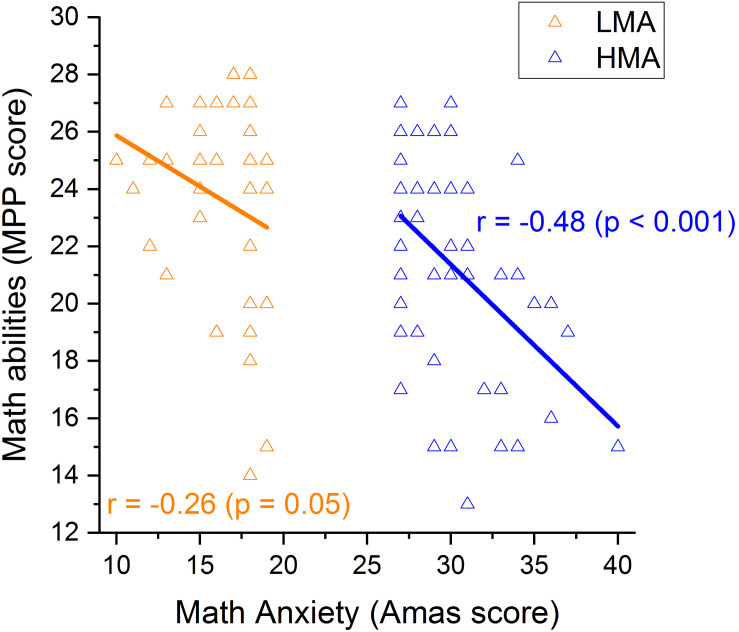
Correlations between math anxiety and math in participants with LMA (orange) and those with HMA (blue).

**FIGURE 4:**
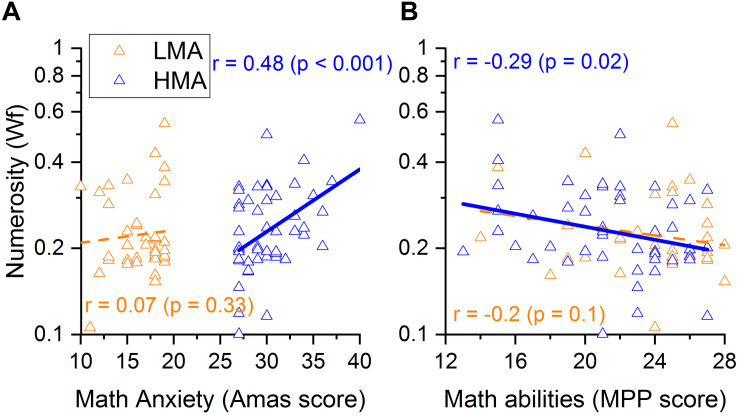
**(A)** Correlations between Numerosity discrimination thresholds and math anxiety or **(B)** math scores for the low math anxiety participants (in orange) and high math anxiety participants (in blue).

Within the HMA group, results demonstrated a significant correlation between MA level and math abilities, with individuals with higher levels of MA having lower math scores (*r* = − 0.479, *p* < 0.001; Figure 3). Moreover, participants with worse numerosity thresholds (higher Wf) also showed higher levels of MA (*r* = 0.48, *p* < 0.001; Figure 4A) and lower math scores (*r* = −0.29, *p* < 0.02; Figure 4B). Interestingly, object size discrimination thresholds were not related to math anxiety level (*r* = −0.065, *p* = 0.33, see Table 2) nor to math scores (*r* = −0.19, *p* = 0.1, see Table 2). Within the HMA group, participants with better performance in the Multiple Object Tracking task (MOT) also had lower math anxiety levels (*r* = −0.255, *p* = 0.04, see Table 2). All the remaining correlations with the MOT task were not statistically significant (*p* > 0.05). Finally, test anxiety did not significantly correlate with any of the aforesaid variables (*p* > 0.05, see Table 2). To further assess the specificity of the link between ANS, MA and math scores, we ran a series of partial correlations taking into account, as covariates, size acuity (WF) and attentional performance (attentional index). These analyses were only run within the HMA group, where bivariate correlations turned out to be statistically significant coefficients. Results of partial correlations revealed that the link between ANS acuity and math anxiety, as well as with math performance, remained statistically significant even when simultaneously controlling for the effects of size acuity, attentional performance and test anxiety [(*r*_(p__artial__)_ = 0.478, *p* < 0.001, *r*_(partial)_ = − 0.3, *p* = 0.019 for math anxiety and math performance respectively].

Within the LMA group, the pattern of correlations changed significantly. Despite math anxiety and math abilities being (marginally) negatively correlated (*r* = − 0.26, *p* = 0.05; Figure 3) within this group, numerosity discrimination thresholds were not related to math-anxiety levels (*r* = 0.07, *p* = 0.33; Figure 4A) nor to math scores (*r* = − 0.20, *p* = 0.1; Figure 4B).

In order to check whether the lack of correlations between numerosity thresholds and MA, and math scores in the group with LMA was due to a difference between subject variance for WF between High and Low anxious individuals, we analyzed and compared variance of numerosity thresholds in the LMA and HMA groups by means of a bootstrap technique (Anobile et al., 2019a). On each of 10,000 iterations (sample-with-replacement), we computed Wf average standard deviation in the LMA and HMA groups separately. We then statistically computed the difference between HMA and LMA by counting the number of times that, in each of the 10,000 iterations, the difference between the average in the HMA sample was higher than the average in the LMA sample (one-tailed *p*-value). The *p*-value was 0.56, suggesting that the lack of correlations described above did not depend on a different level of variance in the data of the two (LMA and HMA) groups. With the same procedure we also excluded a difference in the degree of variability in the MA scores between the two groups (*p* = 0.1).

### Mediation Analysis

**FIGURE 5:**
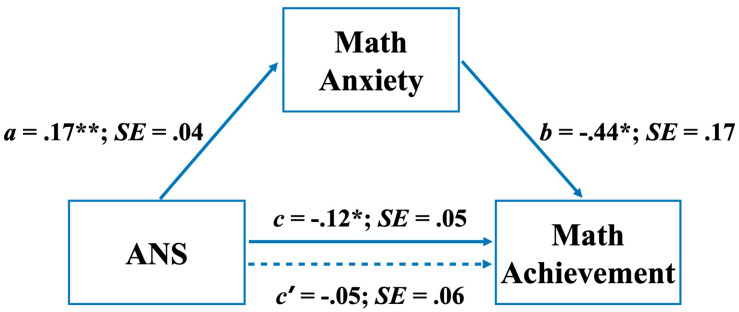
Path coefficients for mediation analysis on achievement; a, b, c, and c′ are unstandardized ordinary least squares (OLS) regression coefficients. *p < 0.05; **p < 0.01.

Given the robust link between numerosity perception (ANS) and math abilities in the group with HMA (see right panel in Figure 4), we explored the nature of this link by measuring the mediating role of MA. For this purpose, we ran a mediation model to derive the total, direct, and indirect effects of ANS on math achievement through MA. As shown in Figure 5, results indicate a significant total effect of ANS on math achievement while the direct effect, their relationship not mediated by MA, was found to be not significant. In contrast, a significant negative indirect effect of ANS on math achievement was found when MA was considered as a mediator. Indeed, the bias-corrected bootstrap 95% CI for the product of these paths (ab) did not include zero (point estimate = -0.08, 95% CI = [-0.1459, -0.0109]), indicating an indirect effect (Preacher and Kelley, 2011).

## Discussion

In the current study, we found that numerosity and object size discrimination thresholds, as well as the ability to attentively track objects in space (MOT), did not differ, on average, between university students with high and low levels of math anxiety. Interestingly, within the high math-anxiety group, numerosity (but not object size) thresholds correlated with both math abilities scores and math- anxiety levels. Crucially, the link between numerosity and math was fully mediated by math-anxiety levels. Overall, our data replicates previous studies on the link between math abilities and numerosity perception but also provided innovative information on the key role that math anxiety plays in such a relationship. Moreover, the fact that math anxiety was found not to be related to size discrimination thresholds, nor to the ability to attentively track objects in space (MOT), strongly suggests that the link between numerosity perception and math-anxiety is not generic but reflects a specific relationship within the numerosity-domain.

Several previous studies have shown that individuals with HMA performed worse on several numerical and mathematical tasks, compared with their low math anxious peers (Ashcraft and Faust, 1994; Maloney et al., 2011). Individuals with lower levels of mathematical skills and high levels of math anxiety show the tendency to avoid situations and careers that require mathematical abilities (Hembree, 1990; Ashcraft, 2002). Given the significant impact of MA on an individual’s quality of life, it is important to better understand its nature. Moreover, to devise successful supporting strategies to reduce the level of anxiety related to math procedures, it might be important to find a predictor or a correlated dimension to MA which could be assessed even before the beginning of school. Some studies suggest that such a dimension might be ANS acuity.

In the current study, we tackled this issue by investigating whether the performance in several perceptual tasks concerning parietal driven magnitude processing (discrimination of stimuli numerosity or size) were related to MA as well as math proficiency. We found that MA is an intermediary factor in the link between math abilities and numerosity perception (ANS acuity) in individuals with HMA. The ANS is considered to have evolutionary roots and it appears very early during development (Starkey et al., 1990; Dehaene et al., 1998). Maloney et al. (2010) suggested that a deficit of basic and core numerical knowledge, such as numerical information, could produce MA (Maloney et al., 2011). By taking into account individuals located in the tails of the MA distribution, a procedure exploited by several previous studies (Maloney et al., 2010; Maloney et al., 2011; Suárez-Pellicioni et al., 2013; Núñez-Peña and Suárez-Pellicioni, 2015; Colomé, 2019), and by considering as a measure of ANS acuity the Weber Fractions (Wf; Piazza et al., 2004, 2010; Halberda et al., 2008; Mazzocco et al., 2011), we found that a significant correlation between ANS precision and MA only exists in HMA groups. Our data shows that individuals with very high levels of MA also have a noisy approximate number sense. Notably, the lack of correlation in the LMA group between these two variables was not due to a difference in variability between the two samples. These results are not just important *per sè*, but also because they are likely to resolve the controversy in the literature about a possible link between MA and ANS precision. For example, Lindskog and Poom (2017) reported that individuals with high levels of MA also show lower ANS precision compared to low mathematics-anxious individuals. However, other studies reported that MA and ANS acuity did not significantly covary in adults (Braham and Libertus, 2018; Dietrich et al., 2015) or in children (Wang et al., 2015; Hart et al., 2016). One possibility is that MA and ANS acuity covaried differently according to the MA level. For example, in the present study a significant correlation between these two dimensions was found just within the group of participants with HMA. On the contrary, by considering all participants as a whole, MA and ANS acuity shows a weaker correlation that turned out to be marginally significant. In other words, ANS precision and MA strongly correlated in the group of HMA individuals but much less in the group of LMA. If so, the statistical significance of the correlation amongst these dimensions, when the two groups are not independently taken into account, depends on the amount of HMA participants and the severity of their anxious levels, variables which robustly differed in the studies reporting conflicting results in the literature.

Our data highlighted another important point: individuals situated in the lower tail of the HMA group performed better in the numerosity task than the individuals situated in the upper tail of the LMA group. This result supports the idea that an “optimum” level of MA might exist which, if exceeded, becomes deleterious not only for math performance (Evans, 2000), but also for discrimination of abstract numerosity. Furthermore, our findings provide supporting evidence to the theory that individuals with a noisy ANS may be more likely to have significant levels of MA. Poor ANS could increase the probability of going through an initial failure and negative learning experience during math education in childhood (Lindskog et al., 2017). One possible explanation of our data is that math abilities and ANS (Weber fraction) are separate (partially independent) predictors of MA, suggesting a bidirectional relationship between MA and math performance, in which a poor ANS induces a low performance in math related tasks and this, in turn, induces MA. This increase in MA might, subsequently, negatively impact math performance, establishing a vicious cycle that dramatically affect an individual’s performance and quality of life.

Math anxiety is strongly correlated with math abilities in individuals with HMA. In line with previous studies, we found that higher levels of MA are linked to lower performance in school or college tests (Hembree, 1990; Ma and Kishor, 1997). MA is at least partly related to fear of failure, so that repeated experiences of failure in mathematics, involving low scores in formal assessments or personal experience of confusion and bewilderment in mathematical activities, may lead to anxiety. Our results are also in line with other studies showing that adults with higher precision in discriminating non-symbolic quantities show higher abilities in math performance (Libertus et al., 2012; Fazio et al., 2014; Lindskog et al., 2017; Schneider et al., 2017; Braham and Libertus, 2018). However, it should be mentioned that, despite many studies which found statistically significant correlations between math abilities and numerosity perception, the literature on this topic is still controversial as other studies report insignificant correlations (Krueger, 1984; Inglis et al., 2011) and the direction of the causal link between ANS and mathematical skills remains highly unclear. While some research suggests that the ANS is a precursor of later mathematical abilities (Gilmore et al., 2010; Piazza, 2010; Anobile et al., 2013; Park and Brannon, 2013) other research failed to find a correlation between ANS precision and mathematical achievements (Krueger, 1984; Inglis et al., 2011; Feigenson et al., 2013; Anobile et al., 2018a). Even if the reasons subtending these discrepancies are still unclear, recent works suggested the important role of the different tests used to assess formal math abilities (Piazza et al., 2010; Lourenco et al., 2012; Anobile et al., 2013; Braham and Libertus, 2018), the numerical ranges used to assess numerosity perception (Anobile et al., 2016a; Anobile et al., 2019a) as well as the age of the participants (Inglis et al., 2011; Anobile et al., 2018a). For example, Braham and Libertus (2018) recently found that students’ ANS acuity did not correlate with their ability to perform mathematical computations in written format, but the correlation occurred with their ability to perform speeded mental arithmetic and quantitative reasoning problems. Similarly, Anobile et al. (2013) found that numerosity thresholds in neurotypical primary school children were related to math tasks requiring the encoding of digit magnitude (e.g., choose the largest among others) but not with those more related to memory (e.g., tables) or transcoding (e.g., number writing or repetition), replicating evidence on dyscalculic children (Piazza et al., 2010). Other recent works suggested that the link between numerosity perception and math is present only for the perception of intermediate numerosity levels and not for very low (Anobile et al., 2019a) or very high (Anobile et al., 2016a) numerous ensembles. The current study makes the general picture even more complicated as we found a significant correlation between math and ANS only among adults with relatively high level of math anxiety. The mathematical test used in the current study, which was developed by Galli et al. (2011), includes 30 multiple-choice questions covering many aspects of arithmetic knowledge, such as probabilistic reasoning, use of fractions, percentages, ratios, calculation, sorting and others. The test, as a whole, is capable of differentiating subjects with low and high MA and also correlates with numerosity thresholds, at least in the high anxiety group. Future studies on larger and more heterogeneous populations than that involved here, could analyze if and which of these 30 items are more specifically related to both anxiety and numerosity perception.

In addition to the controversial literature on the link between numerosity perception and math abilities, an influential recent theory challenged the idea that numerosity can be encoded by a specialized numerical system. This theory suggests that numerosity and other continuous quantities, such as objects sizes, are perceived by a generalized magnitude system (Henik et al., 2017; Leibovich et al., 2017). In the present study we didn’t find a significant correlation between size and numerosity threshold (Weber fractions). Moreover, whilst numerosity WFs were found to be significantly correlated with math scores, the correlation between math performance and size discrimination thresholds turned out in being not significant. These results clearly contradict the generalized magnitude theory and agree with studies suggesting separate mechanisms for the perception of objects’ numerosity and size. Among these, a recent study found similar results, with no correlations between numerosity and size thresholds as well as between numerosity and size sensory adaptation magnitudes, in both children and adults (Anobile et al., 2018b). Regarding the selective link between numerosity and math abilities, Piazza et al. (2013) showed that the exposure of non-schooled indigenous peoples to mathematical knowledge improves the sensitivity to numerosity but not to the size of objects. Similarly, Anobile et al. (2018b) found that discrimination thresholds for numerosity, but not for objects size, is compromised in dyscalculia. Overall, despite being still under debate, our results favor the idea of a specialized numerosity system, specifically linked to math abilities and math anxiety.

We didn’t observe an impairment in the performance of the visual sustained attention task in subjects with HMA, suggesting that they don’t suffer from a general attentional problem despite previous studies in the literature reporting that sustained attention correlates with non-symbolic numerical perception and mathematical skills (Steele et al., 2012; Anobile et al., 2013). Taken together, these results suggest that the link between non-symbolic numerical processing and MA is genuine and does not arise from a generic deficit in the processing of magnitude information or a generic attentional deficit. Even though our approach did not allow us to infer causal connections between the variables we investigated, and the present results cannot be generalized due to the specific sample we chose (students from the Psychology school with un unbalance sampling between male (34%) and female (76%) students), our findings might have important implications in the study of the relationship between ANS and mathematical skills in children with and without mathematical difficulties (e.g., dyscalculia), where MA is meant to play a key role. Indeed, the present results make clear that, in addressing deficits in mathematical performance, low-level aspects such as the ANS acuity as well as high-level aspects as MA have both to be considered. Future research may test the role of MA in the relationship between ANS and mathematical skills in a population of school-age children with a typical development as well as in age-matched subjects affected by dyscalculia, information which would provide a more detailed description of the interplay between MA, ANS and math proficiency.

## Data Availability Statement

The datasets generated for this study are available on request to the corresponding author.

## Ethics Statement

The studies involving human participants were reviewed and approved by the Ethics Commission of the University of Florence. The patients/participants provided their written informed consent to participate in this study.

## Author Contributions

CP and RA developed the study concepts. Testing and data collection were performed by PM. PM, GA, and CP analyzed and interpreted the data. PM, GA, CP, and RA drafted the manuscript. All authors approved the final version of the manuscript for submission and contributed to the study design.

## Conflict of Interest

The authors declare that the research was conducted in the absence of any commercial or financial relationships that could be construed as a potential conflict of interest.

## References

[B1] Anobile G. Arrighi R. Burr D. C. (2019a). Simultaneous and sequential subitizing are separate systems, and neither predicts math abilities. *J. Exp. Child Psychol.* 178 86–103. 10.1016/j.jecp.2018.09.017 30380457

[B2] Anobile G. Arrighi R. Castaldi E. Grassi E. Pedonese L. Moscoso P. A. M. (2018a). Spatial but not temporal numerosity thresholds correlate with formal math skills in children. *Dev. Psychol.* 54 458–473. 10.1037/dev0000448 29239633

[B3] Anobile G. Burr D. C. Gasperini F. Cicchini G. M. (2019b). Near optimal encoding of numerosity in typical and dyscalculic development. *Cortex* 120 498–508. 10.1016/j.cortex.2019.07.009 31520845

[B4] Anobile G. Burr D. C. Iaia M. Marinelli C. V. Angelelli P. Turi M. (2018b). Independent adaptation mechanisms for numerosity and size perception provide evidence against a common sense of magnitude. *Sci. Rep.* 8:13571. 10.1038/s41598-018-31893-6 30206271 PMC6134088

[B5] Anobile G. Castaldi E. Turi M. Tinelli F. Burr D. C. (2016a). Numerosity but not texture-density discrimination correlates with math ability in children. *Dev. Psychol.* 52 1206–1216. 10.1037/dev0000155 27455185 PMC5055099

[B6] Anobile G. Cicchini G. M. Burr D. C. (2016b). Number as a primary perceptual attribute: a review. *Perception* 45 5–31. 10.1177/0301006615602599 26562858 PMC5040510

[B7] Anobile G. Stievano P. Burr D. C. (2013). Visual sustained attention and numerosity sensitivity correlate with math achievement in children. *J. Exp. Child Psychol.* 116 380–391. 10.1016/j.jecp.2013.06.006 23933254

[B8] Ansari D. Lyons I. M. van Eimeren L. Xu F. (2007). Linking visual attention and number processing in the brain: the role of the temporo-parietal junction in small and large symbolic and nonsymbolic number comparison. *J. Cogn. Neurosci.* 19 1845–1853. 10.1162/jocn.2007.19.11.1845 17958487

[B9] Ashcraft M. H. (2002). Math anxiety: personal, educational, and cognitive consequences. *Curr. Direct. Psychol. Sci.* 11 181–185. 10.1111/1467-8721.00196

[B10] Ashcraft M. H. Faust M. W. (1994). Mathematics anxiety and mental arithmetic performance: an exploratory investigation. *Cogn. Emot.* 8 97–125. 10.1080/02699939408408931

[B11] Ashcraft M. H. Kirk E. P. (2001). The relationships among working memory, math anxiety, and performance. *J. Exp. Psychol. Gen.* 130 224–237. 10.1037/0096-3445.130.2.224 11409101

[B12] Ashcraft M. H. Krause J. A. (2007). Working memory, math performance, and math anxiety. *Psychon. Bull. Rev.* 14 243–248. 10.3758/BF03194059 17694908

[B13] Ashcraft M. H. Moore A. M. (2009). Mathematics anxiety and the affective drop in performance. *J. Psychoeduc. Assess.* 27 197–205. 10.1177/0734282908330580 30682125

[B14] Ashcraft M. H. Ridley K. S. (2005). “Math anxiety and its cognitive consequences: a tutorial review,” in *Handbook ok Mathematical Cognition*, ed. Campbell J. I. D. (New York, NY: Psychology Press), 315–327.

[B15] Beilock S. L. Maloney E. A. (2015). Math anxiety: a factor in math achievement not to be ignored. *Policy Insights Behav. Brain Sci.* 2 4–12. 10.1177/2372732215601438

[B16] Braham E. J. Libertus M. E. (2018). When approximate number acuity predicts math performance: the moderating role of math anxiety. *PLoS One* 13:e0195696. 10.1371/journal.pone.0195696 29718939 PMC5931636

[B17] Brainard D. H. (1997). The psychophysics toolbox. *Spat Vis.* 10 433–436. 10.1163/156856897x00357 9176952

[B18] Butterworth B. (1999). *The Mathematical Brain.* New York, NY: Macmillan.

[B19] Butterworth B. Varma S. Laurillard D. (2011). Dyscalculia: from brain to education. *Science* 332 1049–1053. 10.1126/science.1201536 21617068

[B20] Carey E. Hill F. Devine A. Szücs D. (2016). The chicken or the egg? The direction of the relationship between mathematics anxiety and mathematics performance. *Front. Psychol.* 6:1987. 10.3389/fpsyg.2015.01987 26779093 PMC4703847

[B21] Castaldi E. Aagten-Murphy D. Tosetti M. Burr D. Morrone M. C. (2016). Effects of adaptation on numerosity decoding in the human brain. *Neuroimage* 143 364–377. 10.1016/j.neuroimage.2016.09.020 27622396 PMC5139983

[B22] Chang H. Beilock S. L. (2016). The math anxiety-math performance link and its relation to individual and environmental factors: a review of current behavioral and psychophysiological research. *Curr. Opin. Behav. Sci.* 10 33–38. 10.1016/j.cobeha.2016.04.011

[B23] Cicchini G. M. Anobile G. Burr D. C. (2016). Spontaneous perception of numerosity in humans. *Nat. Commun.* 7:12536. 10.1038/ncomms12536 27555562 PMC4999503

[B24] Colomé A. (2019). Representation of numerical magnitude in math-anxious individuals. *Q. J. Exp. Psychol.* 72 424–435. 10.1177/1747021817752094 29359641

[B25] Corbetta M. Shulman G. L. (2002). Control of goal-directed and stimulus-driven attention in the brain. *Nat. Rev. Neurosci.* 3 201–215. 10.1038/nrn755 11994752

[B26] De Smedt B. Verschaffel L. Ghesquière P. (2009). The predictive value of numerical magnitude comparison for individual differences in mathematics achievement. *J. Exp. Child Psychol.* 103 469–479. 10.1016/j.jecp.2009.01.010 19285682

[B27] Dehaene S. (2011). *The Number Sense: How the Mind Creates Mathematics.* Oxford: Oxford University Press.

[B28] Dehaene S. Dehaene-Lambertz G. Cohen L. (1998). Abstract representations of numbers in the animal and human brain. *Trends Neurosci.* 21 355–361. 10.1016/s0166-2236(98)01263-6 9720604

[B29] Dehaene S. Spelke E. Pinel P. Stanescu R. Tsivkin S. (1999). Sources of mathematical thinking: behavioral and brain-imaging evidence. *Science* 284 970–974. 10.1126/science.284.5416.970 10320379

[B30] Dietrich J. F. Huber S. Moeller K. Klein E. (2015). The influence of math anxiety on symbolic and non-symbolic magnitude processing. *Front. Psychol.* 6:1621. 10.3389/fpsyg.2015.01621 26579012 PMC4621307

[B31] Eger E. Sterzer P. Russ M. O. Giraud A. L. Kleinschmidt A. (2003). A supramodal number representation in human intraparietal cortex. *Neuron* 37 719–725. 10.1016/s0896-6273(03)00036-9 12597867

[B32] Embretson S. E. Reise S. P. (2000). *Item Response Theory for Psychologists.* Mahwah, NJ: Lawrence Erlbaum Associates.

[B33] Evans J. (2000). *Adults’ Mathematical Thinking and Emotions: A Study of Numerate Practice.* London: Routledge.

[B34] Fazio L. K. Bailey D. H. Thompson C. A. Siegler R. S. (2014). Relations of different types of numerical magnitude representations to each other and to mathematics achievement. *J. Exp. Child Psychol.* 123 53–72. 10.1016/j.jecp.2014.01.013 24699178

[B35] Feigenson L. Libertus M. E. Halberda J. (2013). Links between the intuitive sense of number and formal mathematics ability. *Child. Dev. Perspect.* 7 74–79. 10.1111/cdep.12019 24443651 PMC3891767

[B36] Ferguson A. M. Maloney E. A. Fugelsang J. Risko E. F. (2015). On the relation between math and spatial ability: the case of math anxiety. *Learn. Individ. Differ.* 39 1–12. 10.1016/j.lindif.2015.02.007

[B37] Galli S. Chiesi F. Primi C. (2011). Measuring mathematical ability needed for “non-mathematical” majors: the construction of a scale applying IRT and differential item functioning across educational contexts. *Learn. Individ. Differ.* 21 392–402. 10.1016/j.lindif.2011.04.005

[B38] Gilmore C. K. McCarthy S. E. Spelke E. S. (2010). Non-symbolic arithmetic abilities and mathematics achievement in the first year of formal schooling. *Cognition* 115 394–406. 10.1016/j.cognition.2010.02.002 20347435 PMC3129629

[B39] Gómez-Velázquez F. R. Berumen G. González-Garrido A. A. (2015). Comparisons of numerical magnitudes in children with different levels of mathematical achievement. An ERP study. *Brain Res.* 1627 189–200. 10.1016/j.brainres.2015.09.009 26385418

[B40] Halberda J. Ly R. Wilmer J. B. Naiman D. Q. Germine L. (2012). Number sense across the lifespan as revealed by a massive Internet-based sample. *Proc. Natl. Acad. Sci. U.S.A.* 109 11116–11120. 10.1073/pnas.1200196109 22733748 PMC3396479

[B41] Halberda J. Mazzocco M. M. Feigenson L. (2008). Individual differences in non-verbal number acuity correlate with maths achievement. *Nature* 455 665–668. 10.1038/nature07246 18776888

[B42] Hart S. A. Logan J. A. Thompson L. Kovas Y. McLoughlin G. Petrill S. A. (2016). A latent profile analysis of math achievement, numerosity, and math anxiety in twins. *J Educ Psychol* 108 181–193. 10.1037/edu0000045 26957650 PMC4779361

[B43] Hayes A. F. (2009). Beyond Baron and Kenny: statistical mediation analysis in the new millennium. *Commun. Monogr.* 76 408–420. 10.1080/03637750903310360

[B44] Hayes A. F. (2013). *INDIRECT Macro for SPSS.* Available online at: http://afhayes.com/spss-sas-and-r-macros-and-code.html

[B45] Hayes A. F. Scharkow M. (2013). The relative trustworthiness of inferential tests of the indirect effect in statistical mediation analysis: does method really matter? *Psychol. Sci.* 24 1918–1927. 10.1177/0956797613480187 23955356

[B46] Hembree R. (1990). The nature, effects, and relief of mathematics anxiety. *J. Res. Math. Educ.* 21 33–46. 10.2307/749455

[B47] Henik A. Gliksman Y. Kallai A. Leibovich T. (2017). Size perception and the foundation of numerical processing. *Curr. Direct. Psychol. Sci.* 26 45–51. 10.1177/0963721416671323

[B48] Hopko D. R. Mahadevan R. Bare R. L. Hunt M. K. (2003). The Abbreviated Math Anxiety Scale (AMAS): construction, validity, and reliability. *Assessment* 10 178–182. 10.1177/1073191103010002008 12801189

[B49] Inglis M. Attridge N. Batchelor S. Gilmore C. (2011). Non-verbal number acuity correlates with symbolic mathematics achievement: but only in children. *Psychon. Bull. Rev.* 18 1222–1229. 10.3758/s13423-011-0154-1 21898191

[B50] Krueger L. E. (1984). Perceived numerosity: a comparison of magnitude production, magnitude estimation, and discrimination judgments. *Percept. Psychophys.* 35 536–542. 10.3758/bf03205949 6483555

[B51] Landerl K. Bevan A. Butterworth B. (2004). Developmental dyscalculia and basic numerical capacities: a study of 8-9-year-old students. *Cognition* 93 99–125. 10.1016/j.cognition.2003.11.004 15147931

[B52] Leibovich T. Katzin N. Harel M. Henik A. (2017). From “sense of number” to “sense of magnitude”: the role of continuous magnitudes in numerical cognition. *Behav. Brain Sci.* 40 e164 10.1017/S0140525X16000960 27530053

[B53] Libertus M. E. Odic D. Halberda J. (2012). Intuitive sense of number correlates with math scores on college-entrance examination. *Acta Psychol.* 141 373–379. 10.1016/j.actpsy.2012.09.009 23098904 PMC3495271

[B54] Lindskog M. Winman A. Poom L. (2017). Individual differences in nonverbal number skills predict math anxiety. *Cognition* 159 156–162. 10.1016/j.cognition.2016.11.014 27960118

[B55] Lourenco S. F. Bonny J. W. Fernandez E. P. Rao S. (2012). Nonsymbolic number and cumulative area representations contribute shared and unique variance to symbolic math competence. *Proc. Natl. Acad. Sci. U.S.A.* 109 18737–18742. 10.1073/pnas.1207212109 23091023 PMC3503215

[B56] Ma X. (1999). A meta-analysis of the relationship between anxiety toward mathematics and achievement in mathematics. *J. Res. Math. Educ.* 30 520–540.

[B57] Ma X. Kishor N. (1997). Assessing the relationship between attitude toward mathematics and achievement in mathematics: a meta-analysis. *J. Res. Math. Educ.* 28 26–47.

[B58] Ma X. Xu J. (2004). The causal ordering of mathematics anxiety and mathematics achievement: a longitudinal panel analysis. *J. Adolesc.* 27 165–179. 10.1016/j.adolescence.2003.11.003 15023516

[B59] MacKinnon D. P. Lockwood C. M. Hoffman J. M. West S. G. Sheets V. (2002). A comparison of methods to test mediation and other intervening variable effects. *Psychol. Methods* 7 83–104. 10.1037/1082-989x.7.1.83 11928892 PMC2819363

[B60] Mackinnon D. P. Lockwood C. M. Williams J. (2004). Confidence limits for the indirect effect: distribution of the product and resampling methods. *Multivar. Behav. Res.* 39:99. 10.1207/s15327906mbr3901_4 20157642 PMC2821115

[B61] Malanchini M. Rimfeld K. Shakeshaft N. G. Rodic M. Schofield K. Selzam S. (2017). The genetic and environmental aetiology of spatial, mathematics and general anxiety. *Sci. Rep.* 7:42218. 10.1038/srep42218 28220830 PMC5318949

[B62] Maloney E. A. (2016). “Math anxiety: causes, consequences, and remediation,” in *Handbook of Motivation at School*, 2 Edn, ed. Miele K. R. W. D. B. (New York, NY: Routledge), 408–423.

[B63] Maloney E. A. Ansari D. Fugelsang J. A. (2011). The effect of mathematics anxiety on the processing of numerical magnitude. *Q. J. Exp. Psychol.* 64 10–16. 10.1080/17470218.2010.533278 21113858

[B64] Maloney E. A. Risko E. F. Ansari D. Fugelsang J. (2010). Mathematics anxiety affects counting but not subitizing during visual enumeration. *Cognition* 114 293–297. 10.1016/j.cognition.2009.09.013 19896124

[B65] Mazzocco M. M. Feigenson L. Halberda J. (2011). Impaired acuity of the approximate number system underlies mathematical learning disability (dyscalculia). *Child Dev.* 82 1224–1237. 10.1111/j.1467-8624.2011.01608.x 21679173 PMC4411632

[B66] Morsanyi K. Busdraghi C. Primi C. (2014). Mathematical anxiety is linked to reduced cognitive reflection: a potential road from discomfort in the mathematics classroom to susceptibility to biases. *Behav. Brain Funct.* 10:31. 10.1186/1744-9081-10-31 25179230 PMC4166027

[B67] Núñez-Peña M. I. Suárez-Pellicioni M. (2014). Less precise representation of numerical magnitude in high math-anxious individuals: an ERP study of the size and distance effects. *Biol. Psychol.* 103 176–183. 10.1016/j.biopsycho.2014.09.004 25224181

[B68] Núñez-Peña M. I. Suárez-Pellicioni M. (2015). Processing of multi-digit additions in high math-anxious individuals: psychophysiological evidence. *Front. Psychol.* 6:1268. 10.3389/fpsyg.2015.01268 26347705 PMC4543779

[B69] Odic D. Libertus M. E. Feigenson L. Halberda J. (2013). Developmental change in the acuity of approximate number and area representations. *Dev. Psychol.* 49 1103–1112. 10.1037/a0029472 22889394 PMC4388157

[B70] Park J. Brannon E. M. (2013). Training the approximate number system improves math proficiency. *Psychol. Sci.* 24 2013–2019. 10.1177/0956797613482944 23921769 PMC3797151

[B71] Parsons S. Bynner J. (2005). *Does Numeracy Matter More?.* London: National Research and Development Centre for adult literacy and numeracy.

[B72] Piazza M. (2010). Neurocognitive start-up tools for symbolic number representations. *Trends Cogn. Sci.* 14 542–551. 10.1016/j.tics.2010.09.008 21055996

[B73] Piazza M. Facoetti A. Trussardi A. N. Berteletti I. Conte S. Lucangeli D. (2010). Developmental trajectory of number acuity reveals a severe impairment in developmental dyscalculia. *Cognition* 116 33–41. 10.1016/j.cognition.2010.03.012 20381023

[B74] Piazza M. Izard V. Pinel P. Le Bihan D. Dehaene S. (2004). Tuning curves for approximate numerosity in the human intraparietal sulcus. *Neuron* 44 547–555. 10.1016/j.neuron.2004.10.014 15504333

[B75] Piazza M. Pica P. Izard V. Spelke E. S. Dehaene S. (2013). Education enhances the acuity of the nonverbal approximate number system. *Psychol. Sci.* 24 1037–1043. 10.1177/0956797612464057 23625879 PMC4648254

[B76] Pinheiro-Chagas P. Wood G. Knops A. Krinzinger H. Lonnemann J. Starling-Alves I. (2014). In how many ways is the approximate number system associated with exact calculation? *PLoS One* 9:e111155. 10.1371/journal.pone.0111155 25409446 PMC4237330

[B77] Pooresmaeili A. Arrighi R. Biagi L. Morrone M. C. (2013). Blood oxygen level-dependent activation of the primary visual cortex predicts size adaptation illusion. *J. Neurosci.* 33 15999–16008. 10.1523/JNEUROSCI.1770-13.2013 24089504 PMC4888977

[B78] Preacher K. J. Hayes A. F. (2008). Asymptotic and resampling strategies for assessing and comparing indirect effects in multiple mediator models. *Behav. Res. Methods* 40 879–891. 10.3758/BRM.40.3.879 18697684

[B79] Preacher K. J. Kelley K. (2011). Effect size measures for mediation models: quantitative strategies for communicating indirect effects. *Psychol. Methods* 16 93–115. 10.1037/a0022658 21500915

[B80] Primi C. Busdraghi C. Tomasetto C. Morsanyi K. Chiesi F. (2014). Measuring math anxiety in Italian college and high school students: validity, reliability and gender invariance of the Abbreviated Math Anxiety Scale (AMAS). *Learn. Individ. Differ.* 34 51–56. 10.1016/j.lindif.2014.05.012

[B81] Primi C. Donati M. A. Chiesi F. Morsanyi K. (2018). Are there gender differences in cognitive reflection? Invariance and differences related to mathematics. *Think. Reason.* 24 258–279. 10.1080/13546783.2017.1387606

[B82] Pylyshyn Z. W. Storm R. W. (1988). Tracking multiple independent targets: evidence for a parallel tracking mechanism. *Spat. Vis.* 3 179–197. 10.1163/156856888x00122 3153671

[B83] Rolison J. J. Morsanyi K. O’Connor P. A. (2016). Can I count on getting better? Association between math anxiety and poorer understanding of medical risk reductions. *Med. Decis. Making* 36 876–886. 10.1177/0272989X15602000 26296620

[B84] Schneider M. Beeres K. Coban L. Merz S. Susan Schmidt S. Stricker J. (2017). Associations of non-symbolic and symbolic numerical magnitude processing with mathematical competence: a meta-analysis. *Dev Sci* 20:e12372. 10.1111/desc.12372 26768176

[B85] Spielberger C. D. Gonzalez H. P. Taylor C. J. Algaze B. Anton W. D. (1978). Examination stress and test anxiety. *Stress Anxiety* 5 167–191.

[B86] Starkey P. Spelke E. S. Gelman R. (1990). Numerical abstraction by human infants. *Cognition* 36 97–127. 10.1016/0010-0277(90)90001-z 2225757

[B87] Starr A. Libertus M. E. Brannon E. M. (2013). Number sense in infancy predicts mathematical abilities in childhood. *Proc. Natl. Acad. Sci. U.S.A.* 110 18116–18120. 10.1073/pnas.1302751110 24145427 PMC3831472

[B88] Steele A. Karmiloff-Smith A. Cornish K. Scerif G. (2012). The multiple subfunctions of attention: differential developmental gateways to literacy and numeracy. *Child Dev.* 83 2028–2041. 10.1111/j.1467-8624.2012.01809.x 22804751

[B89] Suárez-Pellicioni M. Núñez-Peña M. I. Colomé A. (2013). Abnormal error monitoring in math-anxious individuals: evidence from error-related brain potentials. *PLoS One* 8:e81143. 10.1371/journal.pone.0081143 24236212 PMC3827466

[B90] Vukovic R. K. Kieffer M. J. Bailey S. P. Harari R. R. (2013). Mathematics anxiety in young children: concurrent and longitudinal associations with mathematical performance. *Contemp. Educ. Psychol.* 38 1–10. 10.1016/j.cedpsych.2012.09.001

[B91] Wang Z. Hart S. A. Kovas Y. Lukowski S. Soden B. Thompson L. A. (2014). Who is afraid of math? Two sources of genetic variance for mathematical anxiety. *J. Child Psychol. Psychiatry* 55 1056–1064. 10.1111/jcpp.12224 24611799 PMC4636726

[B92] Wang Z. Lukowski S. L. Hart S. A. Lyons I. M. Thompson L. A. Kovas Y. (2015). Is math anxiety always bad for math learning? The role of math motivation. *Psychol. Sci.* 26 1863–1876. 10.1177/0956797615602471 26518438 PMC4679544

[B93] Watson A. B. Pelli D. G. (1983). QUEST: a Bayesian adaptive psychometric method. *Percept. Psychophys.* 33 113–120. 10.3758/bf03202828 6844102

[B94] Young C. B. Wu S. S. Menon V. (2012). The neurodevelopmental basis of math anxiety. *Psychol. Sci.* 23 492–501. 10.1177/0956797611429134 22434239 PMC3462591

